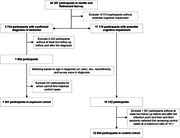# Discontinuity of unmet social support among US adults with potential cognitive impairment before and after the confirmed diagnosis of dementia: an matched ambidirectional cohort study

**DOI:** 10.1002/alz.089518

**Published:** 2025-01-09

**Authors:** Shanquan Chen, Huanyu Zhang, Benjamin R Underwood

**Affiliations:** ^1^ Keppel St, London, London, London United Kingdom; ^2^ Clinical Big Data Research Center, The Seventh Affiliated Hospital, Sun Yat‐sen University, Shenzhen, Shenzhen China; ^3^ University of Cambridge, Cambridge, Cambridgeshire United Kingdom

## Abstract

**Background:**

In the context of heightened attention to dementia, research gaps persist in the seamless integration of clinical and non‐clinical care, including long‐term support. This study aims to examine potential gaps in social support for US adults with cognitive impairment, focusing on the transition before and after a dementia diagnosis.

**Methods:**

In this ambidirectional cohort study, we examined data from the Health and Retirement Survey(HRS) for US adults over 50, using data from 2000 to 2018. The index date was set as either the entry date into HRS or the start of the study, depending on which came later, and follow‐up lasted until death, withdrawal, or study end. Eligibility was limited to participants with potential cognitive impairment during follow‐up, identified through a validated HRS‐based dementia algorithm.

The study divided participants into two groups: the exposure group with a confirmed dementia diagnosis during follow‐up, and the control group, matched by age, sex, race/ethnicity, and survey wave, but without a confirmed diagnosis.

Our primary outcome was unmet social support, defined as reporting physical disability without receiving corresponding social support. We applied a controlled interrupted time series(CITS) model to assess the continuity of unmet social support pre and post‐diagnosis.

**Results:**

From 2000 to 2018, our study involved 1,261 dementia patients in the exposure group and 12,604 in the control group. Following diagnosis, Hispanics showed a significant rise in unmet Basic Activities of Daily Living(BADL) support needs (coef = 0.74, 95%CI[0.03,1.46]), particularly for eating assistance (coef = 1.58, 95% CI[0.17,2.99]). Blacks experienced significant increased unmet BADL needs in toileting (coef = 1.52, 95%CI[0.57,2.47]) and Instrumental ADL (IADL) support (coef = 0.09, 95%CI[0.00,0.17]). Whites saw a significant rise in unmet IADL needs (coef = 0.11,95% CI[0.08,0.14]), especially for making phone calls (coef = 0.83, 95%CI[0.19,1.47]). Further analysis suggested these increases in unmet support were mainly due to a greater rise in disabilities compared to corresponding social support.

**Conclusions:**

Our study identifies a disconnect in the care provided to individuals with dementia before and after their diagnosis. Notably, post‐diagnosis, we observed substantial disparities in unmet social support needs across various racial groups. This highlights the need for more cohesive and equitable care strategies in the dementia care continuum.